# A rare case of pyoderma gangrenosum occurring at the site of laparoscopic port following laparoscopic radical nephrectomy

**DOI:** 10.1002/iju5.12505

**Published:** 2022-07-15

**Authors:** Takuto Ogasawara, Fumimasa Fukuta, Miri Fujita, Hitoshi Tachiki, Tetsuya Shindo, Junji Kato, Hisashi Uhara, Naoya Masumori

**Affiliations:** ^1^ Department of Urology Steel Memorial Hospital Muroran‐shi Japan; ^2^ Department of Urology Sapporo Medical University School of Medicine Sapporo Japan; ^3^ Department of Pathology Steel Memorial Hospital Muroran‐shi Japan; ^4^ Department of Dermatology Sapporo Medical University School of Medicine Sapporo Japan

**Keywords:** laparoscopic surgery, pyoderma gangrenosum, surgical site infection

## Abstract

**Introduction:**

Pyoderma gangrenosum is a rare dermatological disease associated with underlying inflammatory conditions.

**Case presentation:**

A 59‐year‐old man was diagnosed with right renal cancer cT1aN0M0 and laparoscopic right radical nephrectomy was performed. Five days after surgery, he had a high‐grade fever, surgical site flare, and severe pain. At first, we diagnosed surgical site infection and wound dehiscence. Despite treatment for infection, his general condition and dermatological symptoms did not improve. Thereafter, a dermatologist advised us to perform a skin biopsy and blood culture examinations. Finally, the man was diagnosed with pyoderma gangrenosum according to the pathology of the skin biopsy and negative blood culture. After both intravenous administration of predonisolone and a topical corticosteroid, the high‐grade fever and dermatological symptoms improved greatly.

**Conclusion:**

Although pyoderma gangrenosum is a rare disease, we should bear in mind the disease since the treatment strategy is completely different from that for surgical site infection.

**Abbreviations & Acronyms**
CT: computed tomography
DIC: disseminated intravascular coagulation
PG: pyoderma gangrenosum
PT‐INR: prothrombin time‐international normalized ratio

**Keynote message**

Although PG is a rare disease, it is important to keep in mind the possibility of the disease and treatment options after characteristic dermatologic findings.

## Introduction

PG is a rare dermatological disease associated with underlying inflammatory conditions. The mechanism of this rare disease is not well known and its incidence is reported to be 3–10 cases per million persons per year.^1^ It is reported that PG occurs more commonly in patients with systemic diseases such as rheumatoid arthritis and hematologic malignancies.^2^, ^3^ Although the dermatological symptoms are severe in most cases, predonisolone sodium succinate treatment is effective.^4^ Since mechanical irritation can seriously worsen the symptoms, debridement of the diseased site is not recommended.^5^ Since the appearance of PG often mimics surgical site infection and because of the rarity of the disease, it may be difficult to diagnose, especially for urologists. Here we report a case of PG diagnosed at a surgical site after laparoscopic radical nephrectomy, presenting with severe surgical site flare, epidermolysis, and pain.

## Case presentation

**Fig. 1:**
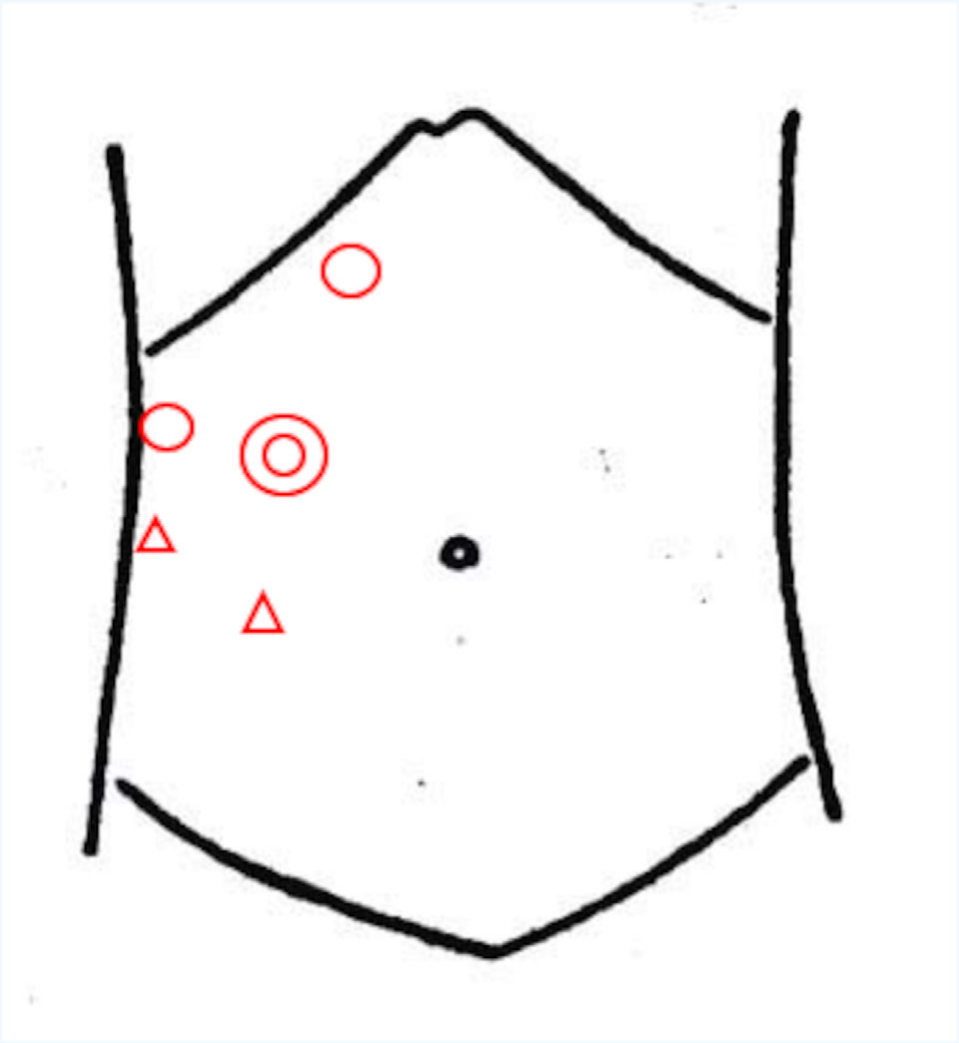
The surgical port site of laparoscopic radical nephrectomy. We used a balloon camera port (◎), two 12 mm instrument ports (○), and two 5 mm instrument ports (▵).

**Fig. 2:**
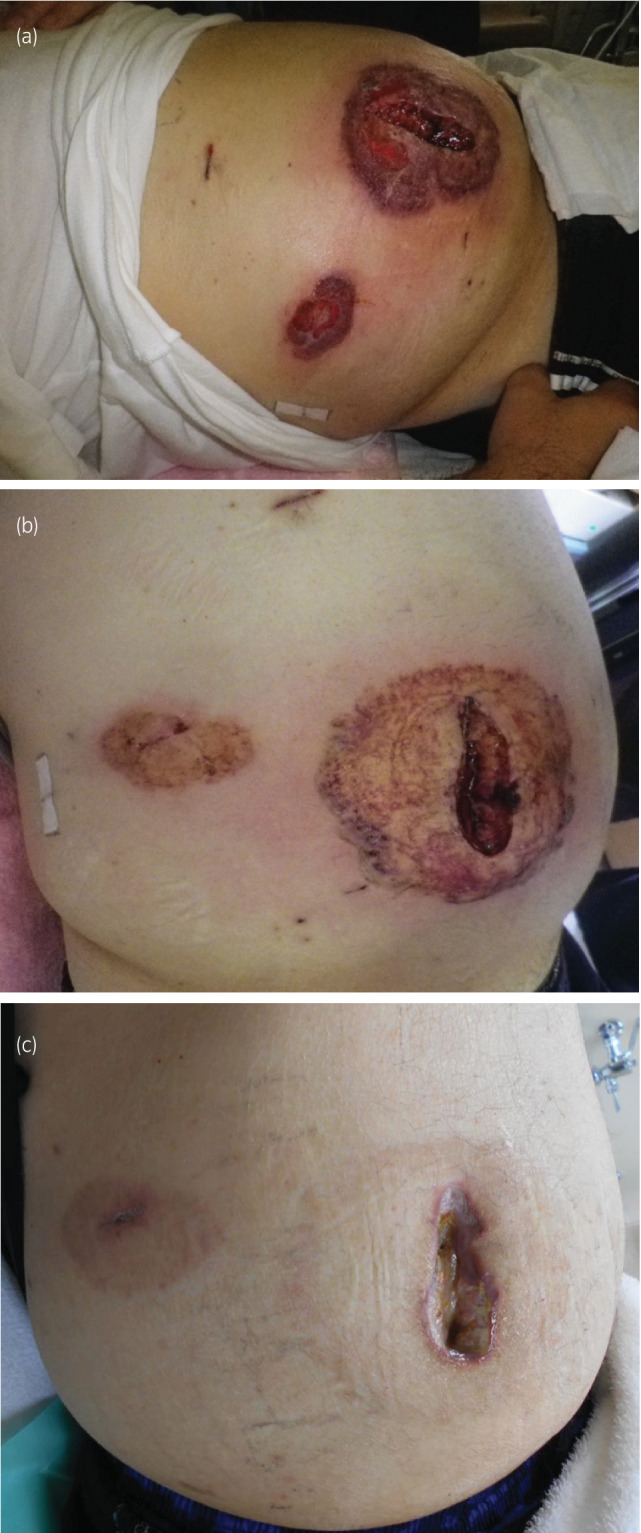
(a) Five days after surgery, the surgical site of the camera port and 12 mm instrument port presented flare, exudate, vesicular bullous lesions, and peeling of the epidermis. (b) Twelve days after surgery, we started both intravenous administration of predonisolone and administration of a topical corticosteroid. The flare of the surgical sites and pain was greatly improved. (c) Three months after discharge, his wounds were greatly improved.

A 59‐year‐old man with an incidental right renal mass was referred to the department of hematology, for follow‐up of follicular lymphoma. After complete remission of the lymphoma, he had been periodically followed up by CT. We diagnosed right kidney cancer cT1aN0M0 by enhanced CT. The past medical history was follicular lymphoma and type 2 diabetes mellitus. There was no surgical history with the skin incision. The tumor was located near the hilum of the kidney, and he underwent laparoscopic right radical nephrectomy without any adverse intraoperative event. The operation time was 210 min (Fig. 1). Three days after surgery, he had a high‐grade fever (>39°C). Physical examination, CT and blood tests (white blood cell 7900/μL, hemoglobin 13.2 g/dL, c‐reactive protein 18.2 mg/dL, procalcitonin 0.23 ng/mL) were performed. Considering the possibility of bacterial infection, we started empiric antibacterial drug treatment (ceftriaxone 2 g/day, vancomycin 0.5 g/day). Five days after the surgery, he complained of fatigue and pain at the surgical site. The camera port and one of the 12 mm instrument ports presented flare, exudate, vesicular‐bullous lesions, and peeling of the epidermis. We initially thought it was a surgical site infection. Therefore, we reopened and checked the wound and did blood culture tests. However, there was little discharge from the wound, which suggested necrosis of the epidermis rather than infection (Fig. 2a).

**Fig. 3:**
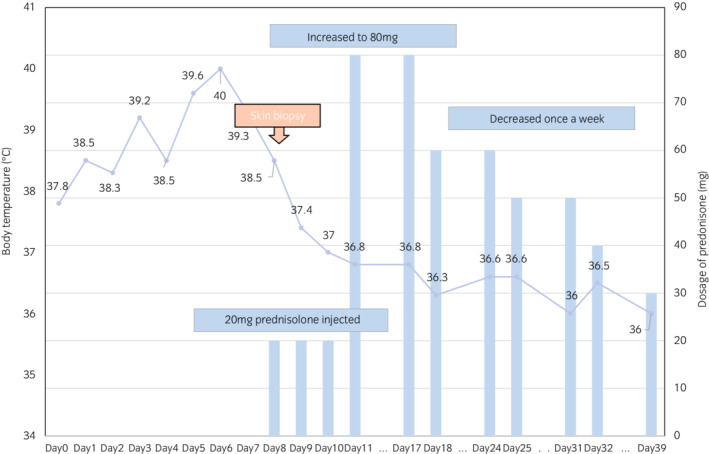
The process of reduction of predonisolone.

**Fig. 4:**
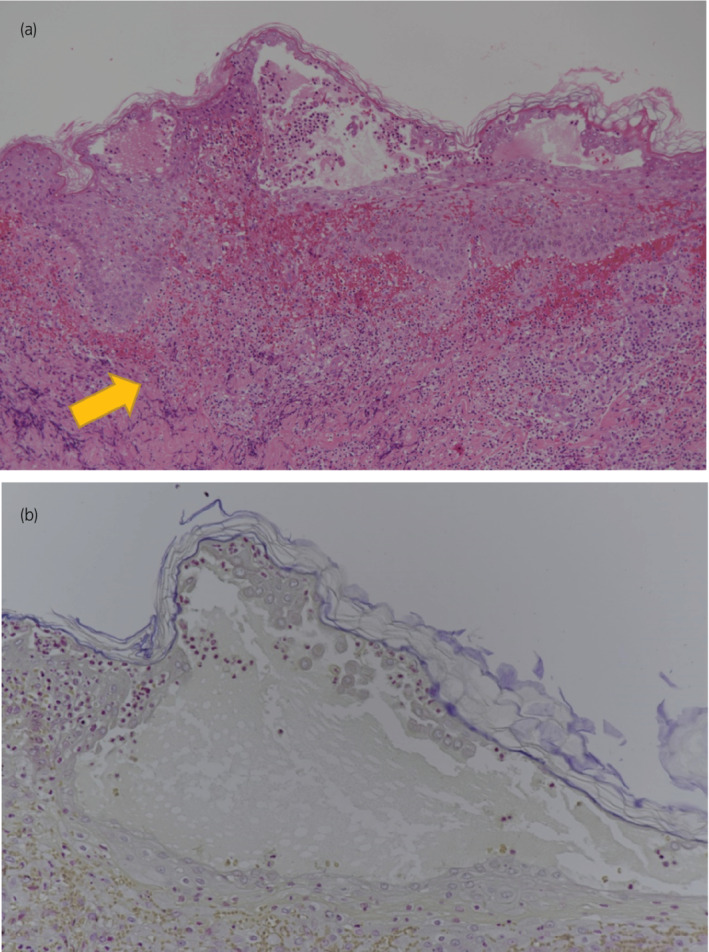
(a) Hematoxylin and eosin stain (×200) shows predominantly neutrophilic infiltrates (yellow), consistent with a diagnosis of PG. (b) Gram stain (×200) is negative for infection.

Regardless of continuous treatment, the high‐grade fever worsened. Consequently, DIC (JAAM DIC diagnostic criteria score: 5) occurred with a high level of serum FDP (11.6 μg/mL), thrombocytopenia (6.2 × 10^4^/μL) and extension of PT‐INR (1.22). Since wound culture was negative and surgical site infection was not plausible, we consulted a dermatologist. The dermatologist suggested a possible diagnosis of PG. We rechecked the wound culture, performed a skin biopsy, and started both intravenous administration of predonisolone (20 mg/day) and administration of a topical corticosteroid. Because we could not completely deny infectious disease, we continued the antibacterial drugs and started relatively low‐dose predonisolone. Thereafter, his condition greatly improved and the second wound culture was negative. Thus, we discontinued the antibacterial drug treatment and raised the predonisolone dose from 20 to 80 mg/day (1 mg/kg/day). His blood test results, general condition, and the dermatological appearance of the port site dramatically improved and we reduced the dosage of intravenous predonisolone to once a week based on consultation with the dermatologist (Fig. 3). Skin biopsy pathology was negative for infection with Gram stains and showed predominantly neutrophilic infiltrates, consistent with a diagnosis of PG (Fig. 4a,b). Thirty‐seven days after surgery, he was discharged from our hospital. In outpatient care, the dermatologist decided on a maintenance predonisolone dosage of 5 mg/day. At 3 months after discharge, the symptoms resulted in great improvement without resuture (Fig. 2c). The pathological diagnosis was right kidney cancer, clear cell carcinoma G2 > G1, INFa v0, pT1a, margin free, and without any abnormality relevant to dermatological findings.

## Discussion

PG is a neutrophilic dermatosis that exhibits predominantly neutrophilic infiltrates without evidence of infection. In 1930, Brunsting *et al*. defined the condition and reported the first PG patient.^6^ PG presents an initial papule, pustule, or nodule after trauma, progressing to painful necrotic ulcers.^7^ The most important features of this disease are an exaggerated response to a minor skin injury or worsening of a wound.^8^ PG is reported to occur in patients who suffer from infection, surgical damage, or, especially, hematologic malignancies.^2^, ^3^ A previous report showed that 20% of PG patients had hematologic disorders.^8^ The patient had a medical history of malignant lymphoma, thus the history may be helpful to diagnose this rare disease. Because there are no useful or specific pathologic features or laboratory markers, the diagnosis of PG is difficult, i.e., it is a “diagnosis of exclusion.” Although no definite uniform criteria for diagnosis exist, Su *et al*. suggested criteria in 2004.^9^ A major criterion and two or more minor criteria are required for diagnosis. “Rapid progression of a cutaneous ulcer with an irregular, violaceous, and ermined border,” and “other causes of cutaneous ulceration have been excluded” are major criteria. Minor criteria were defined as a history suggestive of pathergy or cribriform scarring, systemic diseases associated with PG, histopathologic findings, and treatment response. As treatments for PG, topical corticosteroids and cyclosporine are used, and in severe cases, intravenous administration of methylprednisolone (0.5–1 mg/kg/day) is recommended.^10^

Because surgical intervention can worsen PG, topical debridement is absolutely contraindicated.^11^ In our case, we could not diagnose PG at first, although he had a history of lymphoma and characteristic dermatological findings. The rarity of the disease makes it difficult to diagnose at once. Moreover, PG occurring at the port site of laparoscopic surgery is even rarer. Karthik *et al*. prospectively assessed 570 cases of laparoscopic port site complications.^12^ In their study, port site infection was the most frequent complication (1.8%) and no PG was reported, and there are only several cases reports of PG at the laparoscopic port site following the laparoscopic surgery.^5^, ^13^ In the present case, both the port site and incised site to extract the kidney had the presentation of PG. Thus, PG can occur regardless of the size of the surgical wound.

## Conclusion

We reported a rare case of PG that occurred after laparoscopic radical nephrectomy. Although it is rare, we should bear in mind the disease since the treatment strategy is completely different from that for surgical site infection.

## Author contributions

Takuto Ogasawara: Conceptualization; validation; visualization. Fumimasa Fukuta: Conceptualization; investigation; validation; visualization. Miri Fujita: Conceptualization; visualization. Hitoshi Tachiki: Conceptualization. Tetsuya Shindo: Conceptualization; supervision; validation; visualization. Junji Kato: Conceptualization. Hisashi Uhara: Conceptualization; visualization. Naoya Masumori: Conceptualization; visualization.

## Conflict of interest

The authors declare no conflict of interest.

## Approval of the research protocol by an Institutional Reviewer Board

Not applicable.

## Informed consent

Informed consent was obtained from the patient for publication of this case report.

## Registry and the Registration No. of the study/trial

Not applicable.
